# Differentiating Main-Duct IPMN from Chronic Pancreatitis Using Next-Generation Sequencing of Main Pancreatic Duct Fluid: A Pilot Study †

**DOI:** 10.3390/diagnostics15151964

**Published:** 2025-08-05

**Authors:** Daniel Schmitz, Stefan Prax, Martin Kliment, Felix Gocke, Daniel Kazdal, Michael Allgäuer, Roland Penzel, Martina Kirchner, Olaf Neumann, Holger Sültmann, Jan Budczies, Peter Schirmacher, Frank Bergmann, Jörg-Peter Ritz, Raoul Hinze, Felix Grassmann, Jochen Rudi, Albrecht Stenzinger, Anna-Lena Volckmar

**Affiliations:** 1 Department of Gastroenterology and Infectiology, Helios Kliniken Schwerin, University Campus of Medical School Hamburg, 19055 Schwerin, Germany; stefan.prax@helios-gesundheit.de (S.P.); martin.kliment@helios-gesundheit.de (M.K.); felix.gocke@helios-gesundheoit.de (F.G.); 2 Department of Gastroenterology, Oncology and Diabetology, Theresienkrankenhaus und St. Hedwig-Klinik, 68165 Mannheim, Germany; j.rudi@bbtgruppe.de; 3 Center for Molecular Pathology (CMP), Institute of Pathology Heidelberg (IPH), University Hospital Heidelberg, 69120 Heidelberg, Germanymichael.allgaeuer@med.uni-heidelberg.de (M.A.); roland.penzel@med.uni-heidelberg.de (R.P.); martina.kirchner@med.uni-heidelberg.de (M.K.); albrecht.stenzinger@med.uni-heidelberg.de (A.S.); anna-lena.volckmar@med.uni-heidelberg.de (A.-L.V.); 4 German Cancer Research Center (DKFZ), 69120 Heidelberg, Germany; 5 Department of General and Visceral Surgery, Helios Kliniken Schwerin, University Campus of Medical School Hamburg, 19055 Schwerin, Germany; 6 Institute of Pathology, Helios Kliniken Schwerin, University Campus of Medical School Hamburg, 19055 Schwerin, Germany; 7 Department of Medical Statistics and Epidemiology, Medical School Hamburg, 20457 Hamburg, Germany

**Keywords:** intraductal papillary mucinous neoplasm, pancreatitis, chronic, endoscopic ultrasound-guided fine needle aspiration, high-throughput nucleotide sequencing, prospective study

## Abstract

**Background**: A dilated main pancreatic duct (MPD) ≥ 5 mm can be observed in main-duct IPMNs (MD-IPMN) and chronic pancreatitis (CP); however, distinguishing between the two differently treated diseases can be difficult. Cell-free (cf) DNA in MPD fluid obtained by EUS-guided FNA might help to distinguish MD-IPMN from CP. **Methods**: All patients with a dilated MPD ≥ 5 mm on EUS during the period of 1 June 2017 to 30 April 2024 were prospectively analysed in this single-centre study, with EUS-guided MPD fluid aspiration performed for suspected MD-IPMN or CP in patients who were suitable for surgery. Twenty-two known gastrointestinal cancer genes, including GNAS and KRAS, were analysed by deep targeted (dt) NGS. The results were correlated with resected tissue, biopsy, and long-term follow-up. **Results**: A total of 164 patients with a dilated MPD were identified, of which 30 (18.3%) underwent EUS-guided FNA, with 1 patient having a minor complication (3.3%). Twenty-two patients (mean MPD diameter of 12.4 (7–31) mm) with a definitive, mostly surgically confirmed diagnosis were included in the analysis. Only a fish-mouth papilla, which was present in 3 of 12 (25%) MD-IPMNs, could reliably differentiate between the two diseases, with history, symptoms, diffuse or segmental MPD dilation, presence of calcifications on imaging, cytology, and CEA in the ductal fluid failing to achieve differentiation. However, GNAS mutations were found exclusively in 11 of the 12 (91.6%) patients with MD-IPMN (*p* < 0.01), whereas KRAS mutations were identified in both diseases. **Conclusions**: GNAS testing by dtNGS in aspirated fluid from dilated MPD obtained by EUS-guided FNA may help differentiate MD-IPMN from CP for surgical resection.

## 1. Introduction

In intraductal papillary mucinous neoplasms (IPMNs) of the pancreas, a dilation of the main pancreatic duct (MPD) of 5–9 mm is considered a “worrisome feature” and an MPD diameter of ≥10 mm is considered a “high-risk stigma” that may indicate incipient or manifest transformation to high-grade dysplasia (HGD) or adenocarcinoma of the pancreas [1,2,3]. According to international guidelines, these patients are therefore offered close surveillance or surgery to detect or treat pancreatic cancer at an early tumor stage. However, MPD dilation is not specific to IPMNs, but can also be observed in other pancreatic diseases such as chronic pancreatitis (CP) [4]. Pancreatic duct stones and calcifications are considered typical signs of CP but have also been detected in up to 20.1% (*n* = 33/164) of MD-IPMN cases [5,6,7]. In another study, 19.2% (*n* = 30/156) of preoperatively suspected IPMNs with MPD dilation turned out to be benign pancreatic lesions [8], without the need for surgical treatment. Therefore, it is of the utmost clinical relevance to accurately differentiate MD-IPMNs from other pancreatic diseases such as CP to avoid over- or undertreatment. Fluid from pancreatic cyst lesions (PCLs) obtained by endoscopic ultrasound (EUS)-guided fine-needle aspiration (FNA) provides valuable information about cyst type and malignant transformation [9,10,11]. Meanwhile, mucinous cysts such as branch-duct IPMNs can be reliably differentiated from non-mucinous cysts by detecting KRAS/GNAS mutations in cell-free DNA (cfDNA) of aspirated pancreatic cyst fluid using high-throughput sequencing techniques [9,10,11]. Mutations in other gastrointestinal oncogenes and tumor suppressor genes such as APC, FBXW7, PIK3CA, TP53, MAP2K1, NRAS, AKT1, ERBB2, CTNNB1, BRAF, CDKN2A, NRAS, RNF43, SMAD4, VHL, and NOTCH1 can also be found, but seem to be of less importance in differentiating mucinous from non-mucinous cysts [9,11,12,13]. To our knowledge, DNA sequencing of cfDNA from aspirated MPD fluid using deep targeted next-generation sequencing (dtNGS), which has a detection limit of up to 0.01% variant allele frequency (VAF), has not been reported [14]. Therefore, in this prospective study, we investigated MPD fluid obtained by EUS-guided FNA, in addition to clinical and imaging characteristics, of patients with MPD dilation ≥ 5 mm, as well as analysing 22 known gastrointestinal cancer genes (including KRAS and GNAS) by dtNGS, and performing CEA measurement and cytology.

## 2. Patients and Methods

### 2.1. Study Design and Population

This was a prospective, controlled, single-centre study designed to evaluate the predictive value of specific cancer genes in aspirated pancreatic duct fluid obtained via EUS-guided FNA. The clinical trial is registered under code number NCT03820531 (accessed on 20 December 2020) at ClinicalTrials.gov (https://clinicaltrials.gov). All adult patients who received an EUS of the pancreas between June 2017 and April 2024 were screened for an MPD dilation of ≥5 mm. The following inclusion and exclusion criteria were selected for EUS-guided FNA of fluid from the MPD.

Inclusion criteria: Segmental or diffuse MPD dilation of ≥5 mm. Patients who were classified as sufficiently fit for surgical pancreatic resection. Informed consent was given by the patient. Exclusion criteria: Segmental or diffuse MPD dilation of ≥5 mm combined with a solid pancreatic mass obstructing the MPD (e.g., pancreatic ductal adenocarcinoma), mixed-type IPMN, stenosing papillitis, juxtapapillary diverticulum, or impacted gallstone in the papilla. Repeated EUS-FNA showed the same results of MPD fluid analysis in follow-up. Patient revealed not to be fit for surgery in follow-up or follow-up was later refused by the not-operated patient (no definitive diagnosis could be made). There was not enough material obtained to perform an MPD fluid analysis. In addition, the analysis included four patients with histologically proven metastatic MD-IPMN and four patients with long-standing alcoholic CP as positive controls. Ductal fluid was obtained in patients with known CP during an EUS-guided pancreatic duct drainage.

### 2.2. Ethical Statement

The study was approved by the local ethics committee, Medizinische Ethikkom-mission II, at Mannheim University Hospital on 7 February 2017 (approval number 2016-652N-MA). Informed consent was obtained from all study participants after they had received a verbal explanation and been handed detailed patient information.

### 2.3. Pre-Procedural Workup

Patients included in the study were stratified based on age, gender, history of alcohol abuse and/or smoking, symptoms, coexisting pancreatic calcifications or ductal stones on CT or EUS, endoscopic image of the fish-mouth papilla, and presence of worrisome features or high-risk stigmata according to the revised international consensus Fukuoka guidelines for the management of IPMN of the pancreas [1]. Worrisome features associated with pancreatic cysts, such as cysts > 3 cm, thickened/enlarged cyst walls, and a cyst growth rate of >5 mm/2 years, were omitted.

### 2.4. EUS Procedure

**Figure 1:**
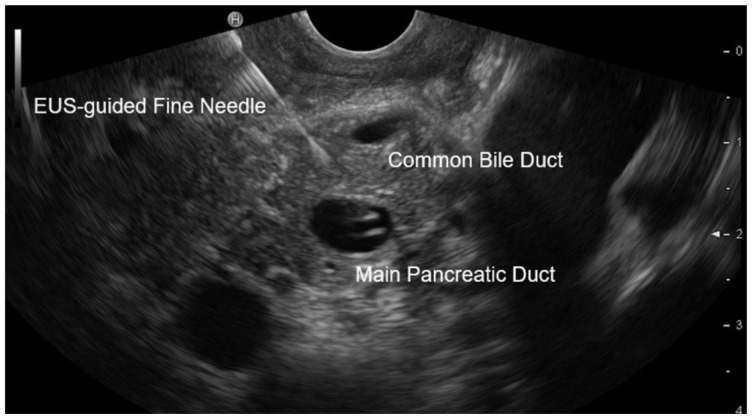
Example of EUS-guided, transgastric FNA of MPD fluid with a 22-gauge needle (Echo Tip^®^ Ultra 3. Cook^®^, Bloomington, IN, USA).

All EUS procedures were performed with a longitudinal Olympus Echo endoscope GF-UCT140T (Tokyo, Japan). EUS-guided transluminal FNA was performed using a Cook Echo Tip^®^ Ultra 3-22G endoscopic ultrasound needle (Bloomington, IN, USA). MPD was only passed once for fine-needle access (Figure 1) to minimize duct injury and to reduce potential access-tract cell seeding. Usually, a minimal aspirate volume of 500 µL is necessary for analysis by dtNGS, and a minimal aspirate volume of 1000 µL is necessary for analysis of CEA level. The remaining content of the puncture needle was used for cytological smear preparation.

### 2.5. Analysis of Aspirated Pancreatic Duct Fluid

The CEA value in MPD fluid was determined using the previously described method [11]. A cut-off value of ≥192 ng/mL was used to identify main-duct IPMN analogue to mucinous cyst lesions [12]. The contents of the EUS puncture needle were applied to one to ten slides. After drying, the slides were stained with haematoxylin and eosin, May-Grünwald Giemsa and Periodic Acid-Schiff. The cytological specimen was assessed according to the actual World Health Organization Reporting System for Pancreaticobiliary Cytopathology [13]. A diagnosis of IPMN was made if IPMN-typical dysplastic epithelial cells were present. A CP diagnosis was made if there were mainly inflammatory cells, such as neutrophil granulocytes, macrophages, and lymphocytes, but no dysplastic epithelial cells. The presence of atypical dysplastic epithelial cells indicated the presence of IPMN-associated high-grade dysplasia or invasive cancer. For DNA sequencing, the aspirated duct was injected into a PAXgene Blood ccDNA Tube (PreAnalytiX, Hombrechtikon, Switzerland), which contained a formaldehyde-free fixative to stabilise nucleated cells. The tube was transferred to the Centre for Molecular Pathology, Institute of Pathology at the University Hospital of Heidelberg, for further workup within 24 h at ambient temperatures by express mail. The procedures for cfDNA extraction, library preparation, semiconductor next-generation sequencing, and data analysis were performed as previously described [14]. DtNGS was performed using a cfDNA Assay (Thermo Fisher Scientific, Waltham, MA, USA) of several known gastrointestinal oncogenes and tumor suppressor genes: AKT1, BRAF, CCND1, CTNNB1, EGFR, ERBB2, GNAS, IDH1/2, KRAS, MAP2K1, NOTCH1, NRAS, PIK3CA, PMS2, RNF43, APC, CDKN2A, FBXW7, PTEN, SMAD4, TP53, and VHL (Supplementary Table S1).

### 2.6. Analysis of Paraffin-Embedded Pancreatic Tissue Samples

Tumor tissue was microdissected to achieve enrichment of MPD epithelial cells. DNA was extracted using the automated Maxwell 16 Research Extraction System (Promega, Madison, WI, USA), following the manufacturer’s guidelines. DNA concentration was measured fluorometrically using a QuBit 2.0 DNA High-Sensitivity Kit (Thermo Fisher Scientific) and by qPCR (RNAseP assay, Thermo Fisher Scientific) to quantify the amount of amplifiable DNA. DtNGS was performed using the same above mentioned cfDNA Assay of twenty-two cancer genes [14].

### 2.7. Statistical Analysis

Analysis of the data was conducted by the Department of Medical Statistics and Epidemiology, Medical School Hamburg, Germany. All statistical calculations were performed using the SAS software, release 9.4 (Cary, NC, USA). To assess the differences between the two diseases (CP and IPMN), we used Fisher’s exact test for count data rather than a chi^2^ test due to the small sample size. We report the observed *p*-value and consider those less than 0.05 to be statistically significant. For qualitative variables, we provide absolute and relative frequencies. For quantitative and approximately normally distributed variables, mean values were calculated. For skewed or ordinal data, the minimum and maximum values are presented.

## 3. Results

### 3.1. Patient Enrolment

**Figure 2:**
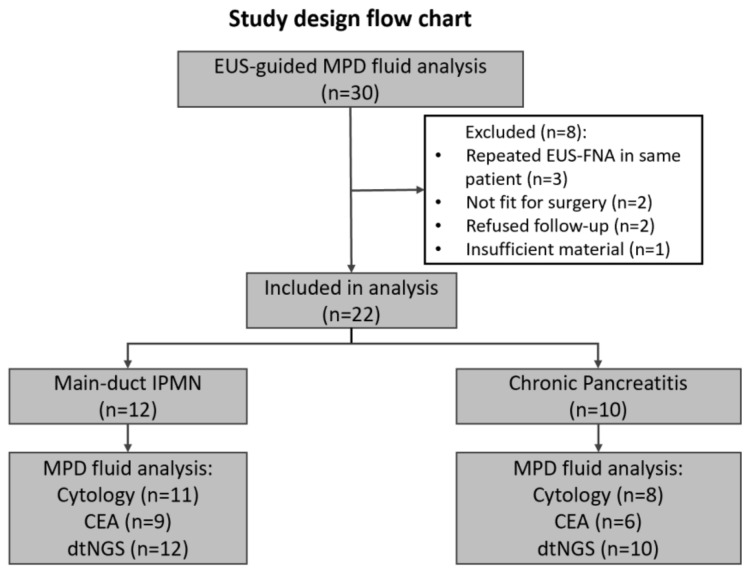
Modified CONSORT flow diagram for study enrolment.

After assessment of the inclusion and exclusion criteria, 30 of 164 (18.3%) patients with a dilated MPD ≥ 5 mm on EUS were prospectively evaluated by EUS-guided MPD fine-needle aspiration. The enrolment process of the patients is shown in the flow chart in Figure 2. Eight patients had to be excluded from further analysis because three of them underwent repeated EUS-guided FNA with the same result as before; two of them were later found to be inoperable (due to comorbidities), and two of them refused follow-up, so the final diagnosis could not be verified. In one patient, the aspirated ductal fluid was insufficient for fluid analysis (<500 µL). In this patient, the EUS-guided FNA of the MPD had to be aborted due to tachycardia and hypotension during propofol sedation, resulting in an overall complication rate of 1 in 30 (3.3%). Therefore, the final analysis included 22 patients.

Four of the twenty-two patients included in the final analysis were diagnosed with MD-IPMN prior to testing and four patients were diagnosed with CP prior to testing. In the remaining fourteen patients, the final diagnosis was clinically and histologically indeterminate (MD-IPMN or CP). Overall, thirteen patients underwent surgical resection (including one patient with intraoperative biopsy) or histological biopsy to clarify the final diagnosis.

### 3.2. Patient Characteristics

The patient characteristics of the 22 patients were similar in MD-IPMN and CP cases, although the patients with MD-IPMN were older and the mean diameter of the MPD was larger in MD-IPMN (Ø 13.5 mm) than in CP (Ø 11.1 mm) cases (Table 1). Histories of smoking and alcohol consumption as well as calcifications of the pancreatic parenchyma and ductal stones were present in both groups. Remarkably, only 3 of the 12 (25%) patients with MD-IPMN had a typical fish-mouth papilla based on endoscopy results.

### 3.3. Analysis of Aspirated Pancreatic Duct Fluid

Comprehensive dtNGS was performed in all 22 patients, while cytology (19 of 22 patients) and CEA (14 of 22 patients) could not be performed in all patients. This was due to an insufficient amount of aspirated fluid for CEA measurement (typically ≥1000 µL required) or a lack of cell detection in the aspirated fluid. Cytology was positive for IPMN in only 2 of 12 patients with MD-IPMN (Table 2). In one sample, the presence of IPMN-associated carcinoma was confirmed through the identification of atypical dysplastic epithelial cells. Conversely, no dysplastic IPMN-typical cells could be identified in the cytology of the MPD aspirate in ten patients with CP. CEA values in MPD fluid were usually higher in MD-IPMN than in CP, although two of the six patients with CP had CEA levels of at least 192 ng/mL, which is the typical cut-off level for mucinous neoplastic PCLs. The main genetic alterations detected occurred in 3 of the 22 analysed GI cancer genes: GNAS, KRAS, and TP53. Interestingly, GNAS mutations were detected in 11 of the 12 patients with MD-IPMN but in none of the patients with CP (*p* < 0.01), whereas KRAS mutations were detected in both diseases, but more frequently in MD-IPMN than in CP (MD-IPMN: *n* = 9/12 and KRAS: *n* = 2/10; *p* = 0.03). The only patient with MD-IPMN without an activating GNAS mutation in the aspirated MPD fluid initially refused surgery and developed biopsy-confirmed MD-IPMN-associated metastatic pancreatic cancer 13 months later.

### 3.4. Presentation of Representative Cases Including Tissue DNA Sequencing

**Figure 3:**
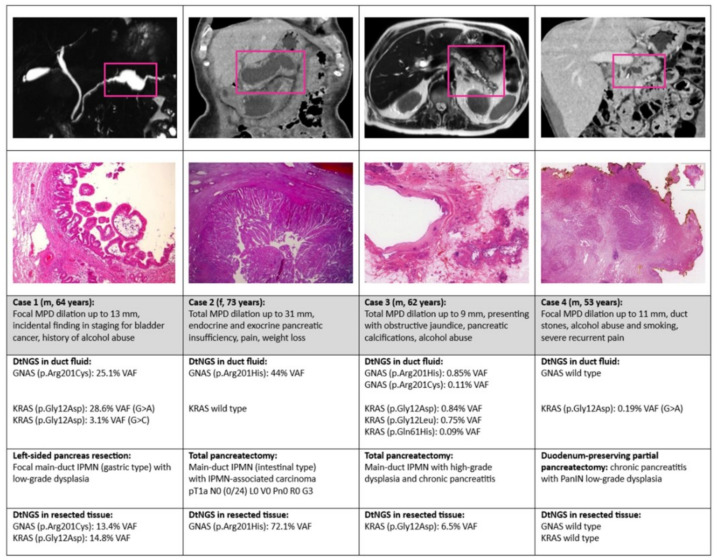
Four representative cases (three with MD-IPMN with LGD, HGD, and pT1a invasive cancer; one with CP with PanIN low-grade dysplasia, 20× magnification) in which dtNGS KRAS/GNAS analysis was performed in aspirated pancreatic duct fluid (obtained by US-guided FNA) and resected pancreatic tissue.

In four representative cases with pancreatic resection (Figure 3), additional DNA sequencing by dtNGS was performed in the resected pancreatic tissue, including the pancreatic duct tissue. In the one patient with HGD in the MD-IPMN and a very low VAF (<1%) of mutated GNAS in the ductal fluid (case 3), no GNAS gene variant could be identified in the resected tissue.

Further results on gastrointestinal cancer gene alterations that did not reach significance to differentiate the two diseases are listed in Supplementary Table S2. The raw data are available in the open-access repository Biostudies: https://www.ebi.ac.uk/biostudies/studies/S-BSST965 (accessed on 11 December 2022).

## 4. Discussion

This prospective pilot study analysed the fluid obtained from the MPD via EUS-guided FNA using cytology, CEA measurement, and dtNGS of cfDNA in order to distinguish between the clinically relevant diagnoses of MD-IPMN and CP.

Patients with MD-IPMN were older and the dilation of MPD was larger in MD-IPMN than in CP. However, the data for both age and MPD diameter showed strong scatter, meaning that age and MPD diameter are not reliable indicators of whether an MD-IPMN or CP is present. Risk factors for CP such as alcohol abuse and smoking, symptoms of pancreatic disease such as abdominal pain, weight loss, diabetes, exocrine pancreatic insufficiency, and obstructive jaundice were present in both groups, although acute (recurrent) pancreatitis was only present in CP. The fact that acute pancreatitis was not present in twelve patients with MD-IPMN might have been due to the small sample size, as acute pancreatitis is a known worrisome feature in IPMN with a sensitivity of 57% and a specificity of 83% [15]. Imaging findings such as diffuse or segmental dilatation of the MPD, as well as pancreatic duct stones and parenchymal calcifications, were common characteristics of both conditions. This finding is consistent with previous studies that identified pancreatic calcifications in up to 20.1% of patients with MD-IPMN [5,6,7]. In contrast, a dilated orifice of the duodenal papilla, known as a fish-mouth papilla, was the only imaging sign that was specific to MD-IPMN and could not be found in any patient with CP. However, it was only present in 25% (*n* = 3/12) of patients with MD-IPMN, which is in line with a reported prevalence of 18–51% in pre-selected patients [16,17]. This may be because only the intestinal subtype appears to be associated with mucus hypersecretion [16]. In addition, an enlarged papillary orifice depends on the subjective assessment of the investigator, even when strict evaluation criteria are used, such as an orifice more than twice the size of a 5F catheter (>3 mm diameter) and mucus protruding from the orifice on visual inspection [16]. Regarding worrisome features and high-risk stigmata, 4/12 patients with MD-IPMN were found to have elevated serum CA 19-9, but none of the patients with CP did. However, this result should be interpreted with caution, as CA 19-9 might be more valuable to distinguish between IPMN with low-grade dysplasia and IPMN with HGD or invasive cancer using a cut-off of ≥133 kU/L [18]. In addition, one study demonstrated that 19% out of the 74 patients with chronic pancreatitis who underwent surgery had elevated CA 19-9 serum levels [19].

IPMN-typical cytology was identified in only two out of twelve (16.7%) patients with MD-IPMN and in none of the patients with CP. The literature on cytology of MPD fluid obtained by EUS-FNA is sparse. In a pilot study involving a highly selective group of 12 patients with various diagnoses, the diagnostic yield was 75% [20]. However, due to the low cellular content of fluid in the MPD, cytology sensitivity is likely to be low, similar to that of mucinous cysts [21].

CEA-levels in MPD fluid varied substantially from 2 to 9370 ng/mL in MD-IPMN and 2 to 1399 ng/mL in CP. If a cut-off value of >192 ng/mL [12], which is used for mucinous cysts, is selected, then only half of patients with MD-IPMN and one-third of patients with CP would test positive for mucinous neoplasia. Therefore, it is unlikely that the CEA value will have sufficient discriminatory power to distinguish between an MD-IPMN and CP, even with a modified cut-off value. This is consistent with the findings of meta-analyses that show the decreasing value of CEA measurement in pancreatic cyst lesions [22,23].

Conversely, GNAS but not KRAS mutations were exclusively found in the MPD fluid of patients with MD-IPMN (91.7%; *n* = 11/12). Analysis of GNAS mutations in MPD fluid obtained via EUS-guided FNA has received little attention in the literature to date. One study conducted extensive DNA mutation analyses of pancreatic duct fluid obtained from 152 patients by cannulating the pancreatic duct during endoscopic retrograde cholangiopancreatography (ERCP) [24]. Four out of six patients (66.7%) with verified MD-IPMN by resection showed a GNAS mutations in pancreatic duct juice. In addition, multivariate analysis indicated that GNAS mutations were associated with dilated MPD in IPMN cases. In contrast, GNAS mutations were found in only 4.5% of the 22 cases with presumed but not resected cases with CP. Considering branch-duct IPMN (BD-IPMN), GNAS mutations are also common, but are rarely detected in other PCLs [25,26]. The detection of activating KRAS mutations in CP in 2 out of 10 patients in this study is consistent with previous publications on KRAS analysis of MPD fluid obtained directly from the MPD or adjacent duodenum by ERCP [27]. According to a systematic review, KRAS mutations were detected in the aspirated MPD fluid (Sanger sequencing) in 24 of 298 (8.1%) patients with CP in studies published up to that point [27]. On the other hand, 50–80% of all IPMNs harbour activating KRAS mutations [28], although the rate is associated with the detection method and generally higher with dtNGS.

Surprisingly, TP53 mutations were observed in the MPD fluid of three out of ten patients with CP. TP53 is a tumour suppressor gene, and mutations in this gene are critical drivers of tumour development and influence the prognosis of pancreatic ductal adenocarcinoma [29]. These mutations can also be found in branch duct-IPMN with HGD or invasive carcinoma [30]. However, TP53 mutations have also been detected in 24 out of 51 (47.1%) tissue samples from cases of severe chronic pancreatitis without associated carcinoma [31]. Therefore, TP53 mutations may not be a reliable biomarker in MPD fluid for distinguishing between MD-IPMN and CP.

Although EUS-guided FNA in PCLs is considered a safe, routine procedure, with a low adverse event rate of 2.6% [32], data on the safety of EUS-guided FNA of MPD fluid are almost non-existent, except for EUS-guided pancreatic duct interventions. Simpson and colleagues [33] reported adverse events in 7/78 (9%) cases of diagnostic EUS-guided FNA of MPD fluid, comprising 5 cases of post-EUS pancreatitis and 2 cases of non-specific abdominal pain. We observed one adverse event of hypotension and tachycardia during sedation in 30 patients (3.3%) who received EUS-guided FNA of MPD fluid. Patients should therefore be informed of the small but relevant risk of post-EUS pancreatitis and abdominal pain. In our experience, the use of a 22G needle represents a good compromise between a low-risk puncture (higher with the 19G needle) and the diagnostic yield of, occasionally difficult to aspirate, mucinous duct fluid (lower with the 25G needle).

This study has several limitations. Firstly, this study was designed as a pilot study involving only twenty-two cases. Therefore, further validation is required in the setting of a multicentre study with a larger sample size. Secondly, although long-term follow-up and a defined cause of CP made the cases highly probable to be cases with CP, not all cases with CP could be verified by surgical resection. Furthermore, we did not identify any patient with CP and associated pancreatic carcinoma in this study, which might have changed the results of the MPD fluid analysis. As previously demonstrated, type 1 autoimmune pancreatitis is associated with an increased risk of pancreatic carcinoma [34]. However, this risk is significantly lower than that associated with MD-IPMN. Thirdly, CEA measurement and cytology could not be performed in all cases. This was due to the large amount of MPD fluid aspirated (>1000 µL) necessary for CEA measurement, and due to the small number of epithelial cells present in the aspirated MPD fluid.

## 5. Conclusions

Activating GNAS oncogene mutation in the fluid of the dilated MPD using DNA-based NGS is a promising biomarker for distinguishing MD-IPMN from CP and is more frequently detectable in MD-IPMN than in fish-mouth papilla. Cytology, KRAS mutation analysis, and CEA (with a cut-off of 192 ng/mL) measurement did not reliably differentiate between MD-IPMN and CP in this pilot study.

## Figures and Tables

**Table 1:** Characteristics of patients with MD-IPMN and CP. ^1^ Received pancreatic enzyme replacement therapy (PERT); ^2^ detected in cross-sectional imaging (EUS or CT); ^3+4^ adapted international consensus Fukuoka guidelines 2017; ^5^ verified by repeated follow-up examinations over five years.

	All *n* = 22	Main-Duct IPMN *n* = 12	Chronic Pancreatitis *n* = 10
Age in years: mean (range)	70.5 (52–82)	74.3 (62–83)	65.9 (52–80)
Sex (m/f/d)	13/9/0	6/6/0	7/3/0
Medical history			
- Alcohol abuse	6	2	4
- Smoking	4	1	3
Symptoms			
- Abdominal pain	15	7	8
- Weight loss	11	9	2
- Diabetes	10	6	4
- Exocrine pancreatic insufficiency ^1^	7	3	4
- Obstructive jaundice	6	5	1
- Acute (recurrent) pancreatitis	3	0	3
- Asymptomatic	2	0	2
Main pancreatic duct dilation			
- Diffuse	15	10	5
- Segmental	7	2	5
MPD diameter in mm: mean (range)	12.4 (7–31)	13.5 (8–31)	11.1 (7–30)
Pancreas with calcification or duct stone (s) ^2^	12	5	7
Fish-mouth papilla	3	3	0
Worrisome features ^3^			
- Main-duct size 5–9 mm	9	3	6
- Abrupt change in caliber of pancreatic duct	7	2	5
- Lymphadenopathy	5	4	1
- Increased serum CA 19-9 (>37 U/L)	4	4	0
- Enhancing mural nodule < 5 mm	0	0	0
High-risk stigmata ^4^			
- Main pancreatic duct > 10 mm	13	9	4
- Obstructive jaundice	6	5	1
- Enhancing mural nodule > 5 mm	5	4	1
Main-duct IPMN			
- Invasive cancer +/− metastases		7	
- HGD		3	
- LGD		2	
Chronic pancreatitis			
- Toxic (alcohol +/− smoking)			4
- Idiopathic			3
- Pancreas divisum			1
- Recurrent biliary pancreatitis			1
- Stenosis of pancreato-jejunal anastomosis			1
Final diagnosis made by			
- Surgery	9	7	2
- Long-term follow-up ^5^	8	1	7
- Biopsy	5	4	1

**Table 2:** Results of cytology, CEA, and dtNGS of KRAS/GNAS in aspirated MPD fluid of patients with MD-IPMN (*n* = 12) and CP (*n* = 10). ^1^ Cytology available in *n* = 19 (overall). ^2^ CEA available in *n* = 14 (overall).

	**All** ***n* = 22**	**Main-Duct IPMN** ***n* = 12**	**Chronic Pancreatitis** ***n* = 10**
Cytology in duct fluid consistent with mucinous neoplasia: *n* (%) ^1^	2/19 (10.5%)	2/11 (18.2%)	0/8 (0.0%)
CEA in duct fluid in ng/mL: mean (range) ^2^	957 (2–9370)	1412 (2–9370)	275 (2–1399)
CEA in duct fluid ≥ 192 ng/mL: *n* (%)	6/14 (42.9%)	4/8 (50.0%)	2/6 (33.3%)
cfDNA-NGS of variant GNAS/KRAS gene in duct fluid: *n* (%)			
- Single or multiple GNAS	11 (50.0%)	11 (91.6%)	0 (0.0%)
- Single GNAS	8 (36.4%)	8 (66.6%)	0 (0.0%)
- Multiple GNAS	3 (13.6%)	3 (25.0%)	0 (0.0%)
- Single or multiple KRAS	11 (50.0%)	9 (75.0%)	2 (20.0%)
- Single KRAS	4 (18.2%)	3 (25.0%)	1 (10.0%)
- Multiple KRAS	7 (31.8%)	6 (50.0%)	1 (10.0%)

## Data Availability

The raw data are available in the open access repository Biostudies: https://www.ebi.ac.uk/biostudies/studies/S-BSST965 (accessed on 11 December 2022).

## Supplementary Materials

The following supporting information can be downloaded at: https://www.mdpi.com/article/10.3390/diagnostics15151964/s1, Supplementary Table S1: The full names and abbreviations of the analysed cancer genes. Supplementary Table S2: Further gastrointestinal cancer gene alterations in MPD fluid detected by dtNGS.

## Abbreviations

The following abbreviations are used in this manuscript:

cfDNA	cell-free deoxyribonucleic acid
CP	chronic pancreatitis
dtNGS	deep targeted next-generation sequencing
ERCP	endoscopic retrograde cholangiopancreatography
EUS	endoscopic ultrasound
HGD	high-grade dysplasia
LGD	low-grade dysplasia
MD-IPMN	main-duct intraductal papillary mucinous neoplasm
MPD	main pancreatic duct
PERT	pancreatic enzyme replacement therapy
PCL	pancreatic cyst lesion
VAF	variant allele frequency
