# Supplementary material for: Differentiating Main-Duct IPMN from Chronic Pancreatitis Using Next-Generation Sequencing of Main Pancreatic Duct Fluid: A Pilot Study
Source: Diagnostics (Basel). 2025 Aug 5;15(15):1964. doi: 10.3390/diagnostics15151964 (PMC12346017; doi:10.3390/diagnostics15151964)
Supplement: Supplementary file 1 [file diagnostics-15-01964-s001.zip › diagnostics-3710235-supplementary.pdf]

Supplementary Table S1: The full names and abbreviations of the analysed cancer genes.

Full name of cancer gene	Abbreviation
**Oncogene**	
V-akt murine thymoma viral oncogene homolog 1	AKT1
B-Rapidly Accelerated Fibrosarcoma Proto-Oncogene Serine/Threonine Kinase	BRAF
B-cell CLL/lymphoma 1, G1/S-specific cyclin D1, U21B31, parathyroid adenomatosis 1	CCND1
Catenin beta 1 is involved in cell adhesion and the regulation of the Wnt signalling pathway	CTNNB1
Epidermal growth factor receptor gene	EGFR
Erythroblastic oncogene B-B2 Receptor tyrosine kinase 2	ERBB2
Guanine nucleotide-binding protein alpha-stimulating activity polypeptide	GNAS
Isocitrate Dehydrogenase 1 and 2, cell metabolism enzyme gene	IDH1/2
Kirsten rat sarcoma viral oncogene homologue	KRAS
Mitogen-activated protein kinase 1	MAP2K1
Neurogenic locus notch homolog protein 1	NOTCH1
Neuroblastoma rat sarcoma viral oncogene homolog	NRAS
Phosphatidylinositol-4,5-bisphosphate 3-kinase catalytic subunit alpha	PIK3CA
Postmeiotic segregation increased 2 homolog, mismatch repair system component	PMS2
RING finger protein 43: regulator of the Wnt signalling pathway	RNF43
**Tumour suppressor gene**	
Adenomatous Polyposis Coli	APC
Cyclin-dependent kinase inhibitor 2A	CDKN2A
F-box and WD repeat domain-containing protein 7	FBXW7
Phosphatase and TENsin homolog	PTEN
Mothers against decapentaplegic homolog 4 (Mad4): mediator of TGF-β-induced tumour suppression	SMAD4
Tumour protein p53	TP53
Von Hippel-Lindau	VHL

Supplementary Table S2: Further gastrointestinal cancer gene alterations in MPD fluid detected by dtNGS.

cfDNA-NGS of further variant cancer genes in duct fluid obtained by EUS-guided FNA: n (%)	All n=22	Main-duct IPMN n=12	Chronic pancreatitis n=10
- Single or multiple TP53	9 (41%)	6 (50%)	3 (30%)
- Single TP53	7 (32%)	4 (33%)	3 (30%)
- Multiple TP53	2 (9%)	2(16%)	0 (0%)
- Single or multiple PIK3CA	3 (14%)	3 (25%)	0 (0%)
- Single PIK3CA	3 (14%)	3 (25%)	0 (0%)
- Multiple PIK3CA	0 (0%)	0 (0%)	0 (0%)
- Single or multiple APC	2 (9%)	2(16%)	0 (0%)
- Single APC	2 (9%)	2(16%)	0 (0%)
- Multiple APC	0 (0%)	0 (0%)	0 (0%)
- Single or multiple FBXW7	2 (9%)	1 (8%)	1 (10%)
- Single FBXW7	1 (5%)	1 (8%)	0 (0%)
- Multiple FBXW7	1 (5%)	0 (0%)	1 (10%)
- Single or multiple CCND1	1 (5%)	1 (8%)	0 (0%)
- Single CCND1	1 (5%)	1 (8%)	0 (0%)
- Multiple CCND1	0 (0%)	0 (0%)	0 (0%)
- Single or multiple CTNNB1	1 (5%)	0 (0%)	1 (10%)
- Single CTNNB1	0 (0%)	0 (0%)	0 (0%)
- Multiple CTNNB1	1 (5%)	0 (0%)	1 (10%)
- Single or multiple BRCA2	1 (5%)	0 (0%)	1 (10%)

- Single BRCA2	1 (5%)	0 (0%)	1 (10%)
- Multiple BRCA2	0 (0%)	0 (0%)	0 (0%)
- Single or multiple PMS2	1 (5%)	0 (0%)	1 (10%)
- Single PMS2	0 (0%)	0 (0%)	0 (0%)
- Multiple PMS2	1 (5%)	0 (0%)	1 (10%)
- Single or multiple PTEN	1 (5%)	1 (8%)	0 (0%)
- Single PTEN	0 (0%)	0 (0%)	0 (0%)
- Multiple PTEN	1 (5%)	1 (8%)	0 (0%)
- Single or multiple IDH1	1 (5%)	1 (8%)	0 (0%)
- Single IDH1	1 (5%)	1 (8%)	0 (0%)
- Multiple IDH1	0 (0%)	0 (0%)	0 (0%)
- Single or multiple IDH2	1 (5%)	0 (0%)	1 (10%)
- Single IDH1	1 (5%)	0 (0%)	1 (10%)
- Multiple IDH1	0 (0%)	0 (0%)	0 (0%)
- Single or multiple SMAD4	1 (5%)	1 (8%)	0 (0%)
- Single SMAD4	1 (5%)	1 (8%)	0 (0%)
- Multiple SMAD4	0 (0%)	0 (0%)	0 (0%)
